# National Data on the Prevalence of Food Insecurity in Australia: Implications for Health Promotion

**DOI:** 10.1002/hpja.70112

**Published:** 2025-10-04

**Authors:** Katherine Kent

**Affiliations:** ^1^ School of Medical, Indigenous and Health Sciences University of Wollongong Wollongong Australia

**Keywords:** equity, food insecurity, health promotion, nutrition policy, public policy, social determinants of health

## 1 Introduction

The Australian Bureau of Statistics (ABS) has released new data showing that one in eight Australian households experienced food insecurity in 2023 [1]. This marks the first nationally representative prevalence estimates collected by the federal government in a decade, and importantly, the first time the ABS has adopted an internationally comparable, validated and sensitive measure of household food insecurity. The findings confirm that food insecurity is a mainstream public health and equity challenge, warranting urgent attention from health promotion professionals, policymakers and researchers.

## 2 Defining Food Insecurity in High‐Income Countries

In high‐income countries, food insecurity is best understood as the inability to consistently acquire adequate, nutritious and culturally acceptable food in socially acceptable ways [2]. Beyond food availability, this definition incorporates the concept of agency—the capacity to exercise choice and autonomy over what foods are eaten, when, and how [3]. Food insecurity therefore includes not only hunger or inadequate dietary intake, but also constrained dietary choices, reliance on food charity and loss of dignity in food acquisition [4]. This broader framing is important for health promotion, as it acknowledges that health and wellbeing are undermined not just by a lack of food, but by erosion of control, cultural appropriateness and social participation [5].

## 3 A Decade Between National Estimates

Until now, national prevalence data on food insecurity in Australia came from the 2011 to 2012 National Nutrition and Physical Activity Survey [6]. At that time, the ABS employed a two‐item measure, which asked whether households had run out of food in the last 12 months and whether they could afford to buy more. In 2011–2012, 4.0% of Australians lived in a household that had run out of food and could not afford to buy more, and 1.5% lived in a household where someone went without food because they could not afford to buy any more [6]. This measure provided a useful but limited snapshot of food insecurity. It was unable to capture the full range of food insecurity experiences, such as anxiety about food, compromised dietary quality or reductions in social acceptability [7].

In contrast, the 2023 ABS data used the 10‐item US Department of Agriculture Household Food Security Survey Module (USDA‐HFSSM) [8]. This instrument has been commonly applied in assessing food insecurity among adults in households in high‐income countries [9], but it does not consider the experience of children, which is captured by the longer 18‐item HFSSM [10]. The tool assesses financial access to food and generates a scale ranging from marginal to severe food insecurity:

1. Marginal food insecurity—anxiety and uncertainty about having enough money for food.
2. Moderate food insecurity—insufficient quality of diet, including reduced variety or nutritional adequacy.
3. Severe food insecurity—insufficient quantity, including skipped meals or smaller portions, and disrupted eating patterns resulting in hunger.

## 4 Why Measuring Food Insecurity Comprehensively Matters

The use of the USDA‐HFSSM means the new ABS prevalence figures are not directly comparable with the 2011–2012 results, and earlier prevalence estimates under‐represented the true extent of the problem [7]. The 2023 ABS survey therefore provides both a more realistic estimate and a richer understanding of the diverse household experiences of food insecurity.

It also allows international benchmarking, though with some caveats. Canada and the United States routinely use the 18‐item USDA HFSSM to monitor food insecurity, which contains the 10 adult items plus an additional eight child‐referenced questions. Australia's 2023 survey applied only the adult 10‐item scale, which means results are directly comparable only for households without children.

With this in mind, Canada has most recently reported 17.8% of households as food insecure [11]. The United States reported 13.5% of households experienced food insecurity over a 1‐year period [12], but unlike Canada and Australia, it does not count marginal food insecurity in its prevalence estimate. On a like‐for‐like basis, Australia's 13.2% is slightly lower than Canada's estimate and similar to the United States. However, these differences may be shaped by methodological decisions as whether marginal insecurity is included, and whether child‐referenced items are asked, can significantly alter prevalence rates [13].

## 5 Monitoring Food Insecurity at Multiple Levels

Although national data remain the cornerstone of policy and international comparison, the Australian evidence base is increasingly multilayered. Several states have instituted surveillance systems that capture the prevalence and determinants of food insecurity with greater granularity. Such monitoring systems have differed in their timing and tools adopted, making interstate prevalence comparisons difficult, but they generate important insights into regional ‘hot spots’ and at‐risk groups, strengthening the evidence base for local action.

In South Australia, prevalence estimates have been generated through multiple surveys using different tools. For example, the Population Health Survey Monitoring System has applied the 18‐item USDA HFSSM since 2020, while the SA Population Health Survey routinely includes a single‐item measure [14]. These quantitative data have been complemented by rich data on lived experiences [15], which together with sector collaboration has informed the development of the SA Food Relief Charter [16] and other state strategies. In Western Australia, the Health and Wellbeing Surveillance System integrated the HFSSM between 2017 and 2021, offering the first state‐wide prevalence estimates, though without adequate regional sampling due to funding limitations [7]. In Victoria, food insecurity data have been collected through various surveys including the Population Health Survey since 2014, directly informing the parliamentary inquiry into food security, the development of healthy food relief guidelines, and other state‐level health initiatives [17]. In Tasmania, academic research [18] and state population‐level health data have been used to shape a state‐led Food Relief to Resilience Strategy [19] and to support recent policy responses to food insecurity such as universal school meal provision. Local and community‐based research [20], further deepens these statistics by capturing lived experiences and guiding tailored local health promotion responses. Taken together, national, state and local monitoring form a layered evidence base that strengthens capacity to design interventions that are both nationally consistent and locally responsive.

## 6 Implications for Health Promotion Activities

Food insecurity is a fundamental health promotion issue. It reflects the interaction between economic, social and environmental determinants of health and has profound impacts on nutrition, physical and mental health and social wellbeing. The new ABS data create opportunities for more targeted and evidence‐informed health promotion strategies [21].

### 6.1 Needs Assessment and Priority Setting

The new data supports better identification of households experiencing marginal or moderate insecurity, not just those in crisis. Health promotion programs can therefore design interventions that prevent households ‘slipping down’ the spectrum, not only those relying on emergency food relief [21]. For example, advocacy for policies that improve food affordability or access to school meal programs can be justified using national‐level prevalence data [22, 23].

### 6.2 Monitoring and Evaluation

While Australia does not yet have a routine food insecurity monitoring and surveillance system, more routine use of the USDA‐HFSSM would allow health promotion practitioners to evaluate the impact of interventions at the population level [24]. If embedded within national health surveys, the measure could track whether changes in income support, food subsidies or local initiatives translate into improved food security [25]. This aligns with health promotion's emphasis on evidence‐based practice and accountability.

### 6.3 Addressing Equity

The ABS has reported preliminary analysis indicating that food insecurity disproportionately affects groups already experiencing disadvantage [1]. More than two in five Aboriginal and Torres Strait Islander households (41%) reported food insecurity, compared with 13% nationally. Single‐parent households reported some of the highest rates, with one in three (34%) affected. Group households, often including students and young workers, reported rates of around 28%. Families with children were more vulnerable (16%) than those without (8%), and nearly one in four households in the lowest income quintile (23%) reported food insecurity, compared with just 3.6% in the highest.

Disaggregated data can highlight these inequities, guiding more tailored and culturally responsive interventions. Health promotion practitioners have a responsibility to ensure programs do not reinforce stigma or rely solely on charitable responses but instead address structural drivers [26].

### 6.4 Policy Advocacy

The Ottawa Charter calls for the creation of supportive environments and the development of healthy public policy. The new ABS data can help to strengthen advocacy for systemic reforms toward increasing income support, implementing universal school meals, improving transport and retail access in rural areas and embedding food security monitoring in national frameworks. Health promotion professionals can use this evidence to argue that food insecurity is not merely a welfare issue, but a determinant of health that requires coordinated, multisectoral action.

### 6.5 Building Research and Practice Partnerships

Finally, the new food insecurity data can provide an opportunity to facilitate stronger partnerships between researchers, practitioners and policymakers. A focus on food insecurity could be integrated with local needs assessments, qualitative research on lived experience and evaluations of interventions. This integration ensures health promotion strategies are both evidence‐based and grounded in community realities.

## 7 Call to Action

For the first time in 10 years, Australia has nationally representative data on household food insecurity. The data confirm that food insecurity affects a significant proportion of households, with implications across nutrition, chronic disease prevention, mental health and equity. Health promotion professionals must seize this moment. The challenge now is not only to acknowledge the scale of food insecurity, but to translate evidence into action. Regular monitoring, equity‐focused interventions and systemic policy change are essential.

The development of a National Food Security Strategy [27] presents a timely opportunity to strengthen Australia's response. To be effective, such a strategy must extend beyond agricultural production to also recognise poverty, nutrition, health and the broader social determinants that shape food access. A joined‐up, cross‐sector approach is required that is informed by robust national monitoring, supported by state and local insights and grounded in the lived experiences of those most affected.

With the right strategies, Australia can ensure that food insecurity is no longer a silent, hidden determinant of poor health, but a preventable challenge at the core of health promotion practice.

## Ethics Statement

The author has nothing to report.

## Conflicts of Interest

The author declares no conflicts of interest.

## Data Availability

Data sharing is not applicable to this article as no datasets were generated or analysed during the current study.
